# Supplementary material for: A non-verbal process dissociation procedure to disentangle explicit from implicit sequence learning
Source: Neurosci Conscious. 2026 Jun 8;2026(1):niag021. doi: 10.1093/nc/niag021 (PMC13245152; doi:10.1093/nc/niag021)
Supplement: SI_R1_niag021 [file si_r1_niag021.pdf]

A non-verbal Process Dissociation Procedure to disentangle explicit from implicit sequence learning

Supplementary information

Instructions

Supplementary Text S1

Original version (french)

Instructions de l'expérience :

« Merci de prendre part à cette expérience ! Voici quelques indications générales. // Pour débuter chaque essai vous devrez cliquer sur une croix en bas de l'écran. // N'hésitez pas à faire de petites pauses au cours de l'expérience : // au moment où cette croix est affichée, pas au milieu d'un essai. // Si vous n'êtes pas sûr d'avoir compris une instruction ou une question, appelez l'expérimentateur. »

Instructions de l'entraînement :

« Instructions // Dans cette première phase d'entraînement, un feedback vous est donné à chaque essai pour vous indiquer si votre réponse était correcte ou incorrecte. // Vous ne recevrez pas d'autres instructions : essayez de découvrir les règles du jeu sur la base de ces feedbacks.»

Instructions à la fin de l'entraînement :

« Vous avez terminé l'entraînement. // A partir de maintenant, il ne vous sera plus indiqué si vos réponses sont correctes ou incorrectes dans cette tâche. // Faites de votre mieux ! Vous verrez votre score à la fin. »

Instructions de la double tâche :

« Dans la phase qui suit, vous allez voir s'afficher une image, que vous devrez mémoriser. // Puis vous cliquerez sur la cible comme d'habitude. // Vous devrez ensuite cliquer sur l'image que vous avez vue précédemment, en la reconnaissant parmi les différentes options proposées. // A partir de maintenant, le feedback correct/incorrect sera à propos de votre réponse dans cette tâche de reconnaissance d'image. »

Instructions du questionnaire :

« Vous allez à présent devoir répondre à 3 questions. // Ces questions portent toutes sur la phase où le cadre était blanc et où un seul rond apparaissait à la fois. Répondez de votre mieux, en utilisant vos propres mots. If it is easier for you, you can answer in english. »

Message final :

« Bravo ! // Votre score est de [pourcentage de réponses correctes en PDP] // Vous avez terminé cette phase. Ne touchez à rien et appelez l'expérimentateur svp. »

Translated version (english)

Experiment instructions:

> « Thank you for taking part in this experiment! Here are some general guidelines. // To start each test you will have to click on a cross at the bottom of the screen. // Do not hesitate to take small breaks during the experiment: // at the moment when this cross is displayed, not in the middle of a trial. // If you are not sure whether you have understood an instruction or question, call the experimenter. »

Instructions at the begining of the training:

> « Instructions // In this first phase of training, feedback is given to you on each trial to tell you whether your answer was correct or incorrect. // You will not receive further instructions: try to discover the rules of the game based on this feedback. »

Instructions at the end of the training:

> « You have completed training. // From now on, you will no longer be shown whether your answers are correct or incorrect in this task. // Do your best ! You will see your score at the end. »

Dual-task instructions:

> « In the following phase, you will see an image displayed, that you will have to memorize. // Then you will click on the target as usual. // You will then have to click on the image that you saw previously, recognizing it among the different options offered. // From now on, the correct/incorrect feedback will be about your answer in this image recognition task. »

Questionnaire instructions:

> « You will now have to answer 3 questions. // These questions all relate to the phase where the frame was white and only one circle appeared at a time. Answer as best you can, using your own words. If it is easier for you, you can answer in English. »

Final message:

> « Congratulations! // Your score is [percentage of correct answers in PDP] // You have completed this phase. Please do not touch anything and call the experimenter. »

Questionnaire

Supplementary Text S2

Participants filled this questionnaire twice, on the computer. The questionnaire consisted of three progressively specific questions, one visible at a time, with no possibility of going back. The first two questions were free-report, prompting participants to express if they observed regular sequences or repeated patterns in the target moves. The last question asked participants to write down the sequences, using the location numbers from a picture displayed along with the text.

Original

Q1 : Avez-vous remarqué quelque chose au cours de la tâche ? Si oui, quoi ?

Q2 : Avez-vous remarqué des régularités dans la position du rond? (Si vous avez indiqué ça à la question précédente, cliquez sur « Suivant ».)

Q3 : Les positions des ronds sur l'écran ne sont pas toujours aléatoires. Il est possible que certaines séquences soient répétées. Pouvez-vous écrire quelles étaient ces séquences en utilisant les chiffres que vous voyez affichés à l'écran ? Vous pouvez en indiquer jusqu'à quatre.

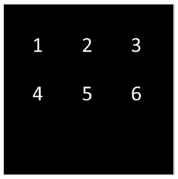

Translated

Q1: Did you notice anything during the task? If so, what?

Q2: Did you notice any regularities in the position of the circle? (If you indicated this in the previous question, click on "Next").

Q3: The positions of the circles on the screen are not always random. It is possible that certain sequences are repeated. Can you write down what these sequences were using the numbers you see on the screen? You can write up to four.

Post-experiment interview

Supplementary Text S3

Interviews were semi-directed and audio-recorded for backup. The experimenter was provided with the questions and indications listed below.

Original version (french)

Merci d'avoir participé à cette expérience !

Nous avons quelques dernières questions à vous poser. Vos réponses à ces questions sont très importantes, prenez votre temps et tentez de répondre au mieux en utilisant vos propres mots.

Q1 : A plusieurs moments de l'expérience vous deviez choisir entre deux cibles (ronds). Il y avait deux règles. Pouvez-vous me dire quelles étaient ces règles ?

Q2 : Rapportez librement toutes vos impressions sur ces moments où vous deviez choisir entre deux cibles.

Q3 : Dans les phases où vous n'aviez pas de choix à faire entre deux cibles, mais où il y avait une seule cible à la fois, avez-vous cherché activement des régularités ?

Si oui : Toujours ou dans certains cas seulement ? Lesquels et pourquoi ? (SINGLE vs. DUAL)

Q4 : A un moment vous deviez mémoriser une image et la retrouver entre deux options. Rapportez librement toutes vos impressions sur cette phase.

Q5 : Y'a-t-il des moments que vous avez trouvé plus difficiles que d'autres ?

Si oui : lesquels et pourquoi ?

Q6 : A propos des questionnaires. Avez-vous eu des difficultés particulières ? (ex : compréhension questions)

Q7 : Avez-vous l'impression que le questionnaire a influencé vos réponses dans la tâche de choix de cible, et inversement ?

Q8 : Pouvez-vous me dire sur quoi l'expérience portait-elle selon vous ?

Autres remarques participant (ex : si fait des suggestions pour que soit plus facile)

Translated verison (english)

Thank you for participating in this experience!

We have a few final questions for you. Your answers to these questions are very important. Please take your time and try to answer as best you can, using your own words.

Q1: At several points in the experiment you had to choose between two targets (circles). There were two rules. Can you tell me what these rules were?

Q2: Freely report all your impressions on these moments when you had to choose between two targets.

Q3: In the phases where you did not have a choice to make between two targets, but where there was only one target at a time, did you actively look for regularities?
If yes : Always or only in certain cases? Which ones and why? (SINGLE vs. DUAL)
Q4: At one point you had to memorize an image and find it between two options. Freely report all your impressions about this phase.
Q5: Are there any moments that you found more difficult than others?
If yes: which ones and why?
Q6: About questionnaires. Did you have any particular difficulties? (e.g. questions comprehension)
Q7: Do you feel that the questionnaire influenced your responses in the target choice task, and vice versa?
Q8: Can you tell me what you think the experiment was about?
Other participant's comments (e.g. if they have suggestions to make it easier)

Stimuli

MTS stimuli

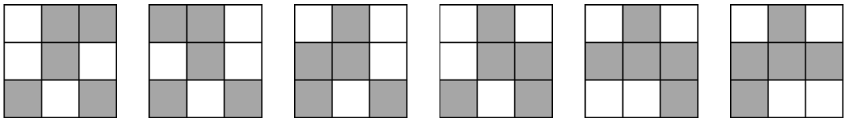

Supplementary Figure S1. *The six stimuli used in the Matching-to-Sample task used as the secondary task intending to disrupt explicit learning.*

Locations

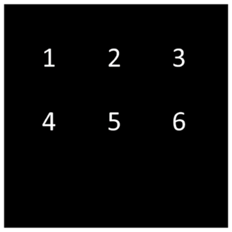

Supplementary Figure S2. *Location numbers for targets displayed on the screen. Note that the numbers are displayed for illustrative purposes only, they were not displayed to participants during the tasks.*

Sequences

Supplementary Table S1. *List of the sequences per set and languages. Numbers refer to the locations on the screen.*

	Seq1	Seq2	Seq3	Seq4
**set1_L1**	3 6 2	4 2 1	5 1 3	6 4 5
**set1_L2**	3 6 1	4 2 5	5 1 2	6 4 3
**set2_L1**	1 3 2	4 5 1	5 6 4	6 2 3
**set2_L2**	1 3 4	4 5 2	5 6 3	6 2 1

Counterbalancing

Subject_num: participant number (i.e. actual order of participation; randomized)
ts1_set, ts1_L, ts2_set, ts2_L: number of the set (set) and language (L) of sequences presented in Phases 1 and 2.
pdp_order: order of the inclusion and exclusion conditions (IE = inclusion then exclusion; EI = exclusion then inclusion).
Q_order: order of presentation of the PDP and the questionnaires (before = questionnaire before PDP; after = questionnaire after PDP).
ts_order: order of presentation of the phases (SD = single-task then double-task; DS = double-task then single-task).

Supplementary Table S2*. Counterbalanced variables for each subject.*

subject	subject num	ts1_set	ts2_set	ts1_L	ts2_L	pdp_order	Q_order	ts_order
**1**	30	1	2	1	1	IE	after	SD
**2**	29	1	2	2	2	IE	after	SD
**3**	14	2	1	1	1	IE	after	SD
**4**	16	2	1	2	2	IE	after	SD
**5**	20	1	2	1	1	EI	after	SD
**6**	22	1	2	2	2	EI	after	SD
**7**	24	2	1	1	1	EI	after	SD
**8**	6	2	1	2	2	EI	after	SD
**9**	1	1	2	1	1	IE	before	SD
**10**	12	1	2	2	2	IE	before	SD
**11**	23	2	1	1	1	IE	before	SD
**12**	32	2	1	2	2	IE	before	SD
**13**	25	1	2	1	1	EI	before	SD
**14**	9	1	2	2	2	EI	before	SD
**15**	8	2	1	1	1	EI	before	SD
**16**	5	2	1	2	2	EI	before	SD
**17**	2	1	2	1	1	IE	after	DS
**18**	17	1	2	2	2	IE	after	DS
**19**	26	2	1	1	1	IE	after	DS
**20**	28	2	1	2	2	IE	after	DS
**21**	13	1	2	1	1	EI	after	DS
**22**	7	1	2	2	2	EI	after	DS
**23**	19	2	1	1	1	EI	after	DS
**24**	27	2	1	2	2	EI	after	DS
**25**	21	1	2	1	1	IE	before	DS
**26**	11	1	2	2	2	IE	before	DS
**27**	31	2	1	1	1	IE	before	DS
**28**	4	2	1	2	2	IE	before	DS
**29**	10	1	2	1	1	EI	before	DS
**30**	18	1	2	2	2	EI	before	DS

**31**	3	2	1	1	1	EI	before	DS
**32**	15	2	1	2	2	EI	before	DS

Model outputs

Supplementary Tables S3

Tasks performance as a function of experimental conditions

Learning effects: Grammatical blocks (1-7)* Task condition

	**Dependent variable**		
Predictors	*Estimates*	*CI*	*p*
(Intercept)	621.04	587.35 – 654.72	**<0.001**
block	-14.99	-21.16 – -8.83	**<0.001**
ts cond [dual]	-7.68	-45.84 – 30.48	0.692
Block × ts cond [dual]	2.31	-6.41 – 11.02	0.603
**Random Effects**			
σ^2	8436.76		
$\tau_{00\ subject\_id}$	3367.31		
ICC	0.29		
N $_{subject\_id}$	32		
Observations	384		
Marginal R^2 / Conditional R^2	0.068 / 0.334		

Learning effects: Block 1 vs. Block 7 * Task condition

	**Dependent variable**		
Predictors	*Estimates*	*CI*	*p*
(Intercept)	653.24	611.03 – 695.45	**<0.001**
block [7]	-124.71	-179.58 – -69.84	**<0.001**
ts cond [dual]	-25.77	-80.64 – 29.10	0.354

block × ts cond [dual]	29.81	-47.78 – 107.40	0.448
**Random Effects**			
σ^2	12290.89		
τ_{00} subject_id	2259.32		
ICC	0.16		
N subject_id	32		
Observations	128		
Marginal R^2 / Conditional R^2	0.177 / 0.305		

Violation effects: Grammatical block 4 vs. Violation block 5 & Grammatical block 6 * Task condition

		Dependent variable	
Predictors	*Estimates*	*CI*	*p*
(Intercept)	507.10	458.57 – 555.63	**<0.001**
block [5]	226.70	163.31 – 290.09	**<0.001**
block [6]	60.86	-2.53 – 124.25	0.060
ts cond [dual]	32.08	-31.31 – 95.47	0.319
block × ts cond [dual]	-125.16	-214.81 – -35.51	**0.006**
block × ts cond [dual]	-50.92	-140.57 – 38.73	0.264
**Random Effects**			
σ^2	16517.60		
τ_{00} subject_id	2843.25		
ICC	0.15		
N subject_id	32		
Observations	192		
Marginal R^2 / Conditional R^2	0.232 / 0.345		

PDP: Sequence type * Task condition

	Dependent variable		
Predictors	*Estimates*	*CI*	*p*
(Intercept)	31.38	21.96 – 40.80	**<0.001**
seq type [inclu]	50.13	36.81 – 63.45	**<0.001**
ts cond [dual]	7.55	-5.77 – 20.87	0.264
seq type [inclu] × ts cond [dual]	-18.36	-37.20 – 0.48	0.056
**Random Effects**			
σ^2	724.69		
τ_{00} subject_id	0.00		
N subject_id	32		
Observations	128		
Marginal R^2 / Conditional R^2	0.380 / NA		

Tasks performance as a function of verbal reports

Learning effects: Grammatical blocks (1-7)* Sequence report

	Dependent variable		
Predictors	*Estimates*	*CI*	*p*
(Intercept)	634.70	603.05 – 666.34	**<0.001**
block	-19.44	-24.88 – -14.01	**<0.001**
seq report [Seq-Unreported]	-46.67	-88.93 – -4.41	**0.031**
block × seq report [Seq-Unreported]	14.94	6.08 – 23.81	**0.001**
**Random Effects**			
σ^2	8188.82		
τ_{00} subject_id	3284.81		
ICC	0.29		

$N_{subject\_id}$	32
Observations	384
Marginal R^2 / Conditional R^2	0.089 / 0.350

Learning effects: Block 1 vs. Block 7 * Sequence report

	Dependent variable		
Predictors	*Estimates*	*CI*	*p*
(Intercept)	664.68	626.24 – 703.12	**<0.001**
block [7]	-157.67	-204.19 – -111.16	**<0.001**
seq report [Seq-Unreported]	-64.87	-123.13 – -6.61	**0.029**
block [7] × seq report [Seq-Unreported]	127.65	51.69 – 203.61	**0.001**
**Random Effects**			
σ^2	11043.29		
$\tau_{00\ subject\_id}$	2645.18		
ICC	0.19		
$N_{subject\_id}$	32		
Observations	128		
Marginal R^2 / Conditional R^2	0.226 / 0.376		

Violation effects: Grammatical block 4 vs. Violation block 5 & Grammatical block 6 * Sequence report

	Dependent variable		
Predictors	*Estimates*	*CI*	*p*
(Intercept)	493.51	449.35 – 537.66	**<0.001**
block [5]	242.54	188.06 – 297.01	**<0.001**
block [6]	61.14	6.67 – 115.62	**0.028**
seq report [Seq-Unreported]	79.02	11.84 – 146.20	**0.021**

block [5] × seq report [Seq-Unreported]	-209.12	-298.08 – -120.17	**<0.001**
block [6] × seq report [Seq-Unreported]	-68.65	-157.60 – 20.31	0.130
**Random Effects**			
σ^2	15247.43		
τ00 subject_id	3187.46		
ICC	0.17		
N subject_id	32		
Observations	192		
Marginal R^2 / Conditional R^2	0.270 / 0.396		

PDP-Reported: Sequence type * Sequence report

	m_bysuj		
Predictors	*Estimates*	*CI*	*p*
(Intercept)	0.06	0.01 – 0.12	**0.013**
seq type [inclu]	0.87	0.80 – 0.94	**<0.001**
seq report combined [Seq-Unreported]	0.43	0.32 – 0.53	**<0.001**
seq type [inclu] × seq report combined [Seq-Unreported]	-0.74	-0.89 – -0.59	**<0.001**
**Random Effects**			
σ^2	0.02		
τ00 subject_id	0.00		
N subject_id	19		
Observations	76		
Marginal R^2 / Conditional R^2	0.888 / NA		

PDP individual scores

Supplementary Table S4. *PDP individual scores: Khi-tests on the difference in grammatical target choice between inclusion and exclusion conditions for each participant as a function of verbal reports. Note that only PDP-Reported and PDP-Unreported participants' performance is presented in this table (N=26). Participants with incomplete report of the PDP rules (N=6) were discarded from PDP analyses.*

subject_cond	PDP_report	seq_report	exclu	inclu	diff	khi_p
9S	PDP-Unreported	Seq-Reported	0.67	0.29	-37.50	0.061
9D	PDP-Unreported	Seq-Reported	0.62	0.33	-29.17	0.144
10S	PDP-Unreported	Seq-Reported	0.67	0.46	-20.83	0.336
5D	PDP-Unreported	Seq-Unreported	0.67	0.54	-12.50	0.577
18D	PDP-Reported	Seq-Unreported	0.79	0.71	-8.33	0.739
7D	PDP-Reported	Seq-Unreported	0.58	0.50	-8.33	0.695
16D	PDP-Unreported	Seq-Unreported	0.67	0.67	0.00	1.000
18S	PDP-Reported	Seq-Unreported	0.46	0.46	0.00	1.000
27S	PDP-Unreported	Seq-Unreported	0.46	0.46	0.00	1.000
3D	PDP-Reported	Seq-Reported	0.46	0.46	0.00	1.000
8D	PDP-Reported	Seq-Unreported	0.67	0.67	0.00	1.000
27D	PDP-Unreported	Seq-Unreported	0.46	0.50	4.17	0.835
28D	PDP-Reported	Seq-Unreported	0.54	0.58	4.17	0.847
10D	PDP-Unreported	Seq-Unreported	0.46	0.54	8.33	0.683
16S	PDP-Unreported	Seq-Unreported	0.71	0.79	8.33	0.739
22D	PDP-Unreported	Seq-Unreported	0.46	0.54	8.33	0.683
26D	PDP-Unreported	Seq-Unreported	0.58	0.67	8.33	0.715
26S	PDP-Unreported	Seq-Unreported	0.46	0.54	8.33	0.683
21D	PDP-Reported	Seq-Unreported	0.46	0.58	12.50	0.549
22S	PDP-Unreported	Seq-Reported	0.46	0.62	16.67	0.433
30S	PDP-Reported	Seq-Reported	0.42	0.58	16.67	0.414
6D	PDP-Reported	Seq-Unreported	0.54	0.75	20.83	0.369
5S	PDP-Unreported	Seq-Unreported	0.38	0.62	25.00	0.221
31D	PDP-Reported	Seq-Unreported	0.21	0.58	37.50	0.039
30D	PDP-Reported	Seq-Reported	0.21	0.71	50.00	0.011
14D	PDP-Reported	Seq-Unreported	0.17	0.75	58.33	0.003
32D	PDP-Reported	Seq-Reported	0.12	0.75	62.50	0.001
8S	PDP-Reported	Seq-Reported	0.21	0.83	62.50	0.003
15S	PDP-Reported	Seq-Reported	0.08	0.96	87.50	0.000

subject_cond	PDP_report	seq_report	exclu	inclu	diff	khi_p
29D	PDP-Reported	Seq-Reported	0.08	0.96	87.50	0.000
14S	PDP-Reported	Seq-Reported	0.04	0.96	91.67	0.000
15D	PDP-Reported	Seq-Reported	0.08	1.00	91.67	0.000
28S	PDP-Reported	Seq-Reported	0.00	0.92	91.67	0.000
12S	PDP-Reported	Seq-Reported	0.04	1.00	95.83	0.000
25S	PDP-Reported	Seq-Reported	0.00	0.96	95.83	0.000
31S	PDP-Reported	Seq-Reported	0.04	1.00	95.83	0.000
6S	PDP-Reported	Seq-Reported	0.04	1.00	95.83	0.000
7S	PDP-Reported	Seq-Reported	0.04	1.00	95.83	0.000
12D	PDP-Reported	Seq-Reported	0.00	1.00	100.00	0.000
1D	PDP-Reported	Seq-Reported	0.00	1.00	100.00	0.000
1S	PDP-Reported	Seq-Reported	0.00	1.00	100.00	0.000
20D	PDP-Reported	Seq-Reported	0.00	1.00	100.00	0.000
20S	PDP-Reported	Seq-Reported	0.00	1.00	100.00	0.000
21S	PDP-Reported	Seq-Reported	0.00	1.00	100.00	0.000
23D	PDP-Reported	Seq-Reported	0.00	1.00	100.00	0.000
23S	PDP-Reported	Seq-Reported	0.00	1.00	100.00	0.000
25D	PDP-Reported	Seq-Reported	0.00	1.00	100.00	0.000
29S	PDP-Reported	Seq-Reported	0.00	1.00	100.00	0.000
2D	PDP-Reported	Seq-Reported	0.00	1.00	100.00	0.000
2S	PDP-Reported	Seq-Reported	0.00	1.00	100.00	0.000
32S	PDP-Reported	Seq-Reported	0.00	1.00	100.00	0.000
3S	PDP-Reported	Seq-Reported	0.00	1.00	100.00	0.000

Individual-level exploratory analyses

Supplementary Text S4

Identifying successful sequence learners. To detect decreases in RTs with exposure, we analyzed participant-level RTs from Blocks 1–4 (before violation in Block 5). For each participant and phase, we fitted three models: 1/ a no-change model, RT ~ β; 2/ a linear-trend, RT ~ $\beta + \alpha$ * block_number; and 3/ a single change-point (step) model, RT ~ β_pre for blocks $\leq c$ and $RT \sim \beta\_post$ for blocks > c, c being the change point. This final model was inspired by Barth et al. (2025),

but differed in the approach used to identify RT drops: first, we tested the three possible change points (between Blocks 1 and 2, 2 and 3, and 3 and 4) and kept the one with the lowest Akaike Information Criterion (AIC). Then, we compared the two RT change models against the no-change model, with an Anova. If both models won against the null model, we retained the one with the lower AIC. Using this procedure, participants were classified as "linear learner" (linear wins and $\alpha < 0$), "RT-drop learner" (change-point wins and $\beta\_post < \beta\_pre$), or "non-learner". Model comparisons indicated that RT-drop patterns were more frequent than linear trends, accounting for 40% of Seq-Reported cases and 33.3% of Seq-Unreported cases (vs. 30% and 12.5%, respectively; see Supplementary Figure S3). Both RT-drop and linear models were associated with larger RT decreases in Seq-Reported than in Seq-Unreported cases.

To assess response slowing to violations, for each participant and phase, we fitted and compared two models: a model including a block effect (violation Block 5 vs. preceding Block 4) and a null model. Recovery was assessed by comparing a model including a block effect (violation Block 5 vs. subsequent Blocks 6 and 7) with a null model. Participants were considered to exhibit a violation effect only if both block-effect models outperformed the corresponding null models. Seq-Reported cases showed a response slowdown followed by recovery in 67.5% of instances, whereas this pattern was observed in only 20.8% of Seq-Unreported cases and was associated with a smaller-magnitude slowdown (368 vs. 128ms). Note that a small number of Seq-Reported cases exhibited a delayed recovery, with RTs being significantly lower in Block 7—but not in Block 6—relative to Block 5, a pattern not observed in Seq-Unreported cases. Although speculative, this could reflect a transient post-violation slowdown associated with sequence awareness, which may merit further investigation.

PDP task performance in successful sequence learners. When considering only successful sequence learners, the Seq-Reported group (N = 19) showed particularly clear-cut PDP performance: on average, participants selected the grammatical target on 99% of inclusion trials and only 0.2% of exclusion trials. In contrast, the Seq-Unreported group (N = 5) selected the grammatical target on 58% of inclusion trials and 56% of exclusion trials. Thus, on average, Seq-Unreported participants appeared to select the grammatical target slightly above chance level in both conditions, a pattern consistent with PDP predictions under an implicit learning account. However, this observation remains anecdotal given the very small sample size.

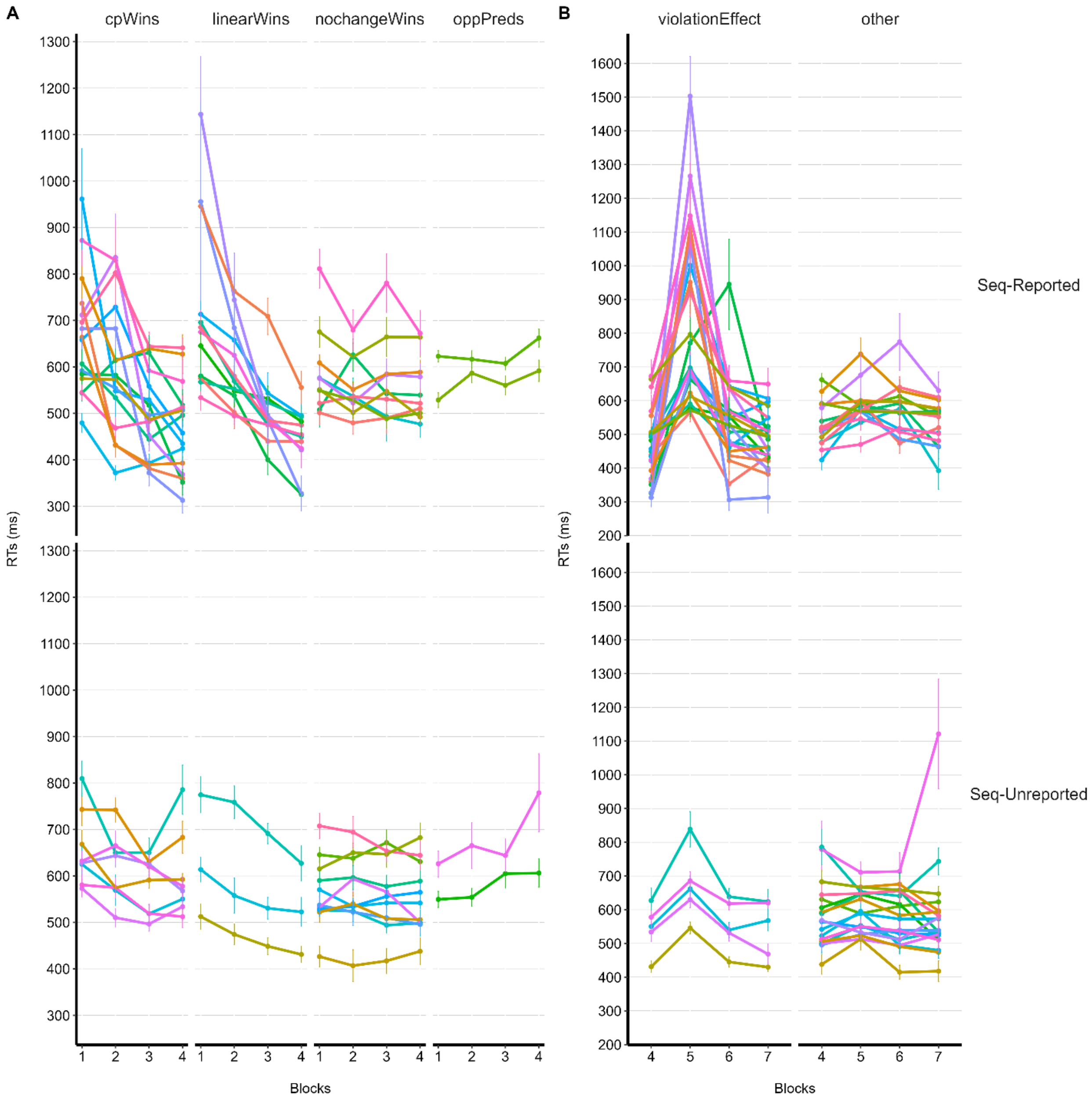

Supplementary Figure S3. *Results of the Serial Reaction Time task split by sequence report and best model fit: mean response times on the third target as a function of block. Left panel illustrates RTs curves for the model fits on the exposure phase (Blocks 1-4): change-point (or RT-drop) model, linear model, no-change model, opposite to predictions. Right panel illustrates RTs curves for the model fits on the violation-recovery phase (Blocks 4-7): violation-effect model (figuring both a significant increase in RTs on the violation block followed by a significant decrease) and others (i.e., where either increase or decrease was detected, or none of the two). See manuscript for model formulas. Each line represents a participant / condition (single or dual). Error bars represent SE.*

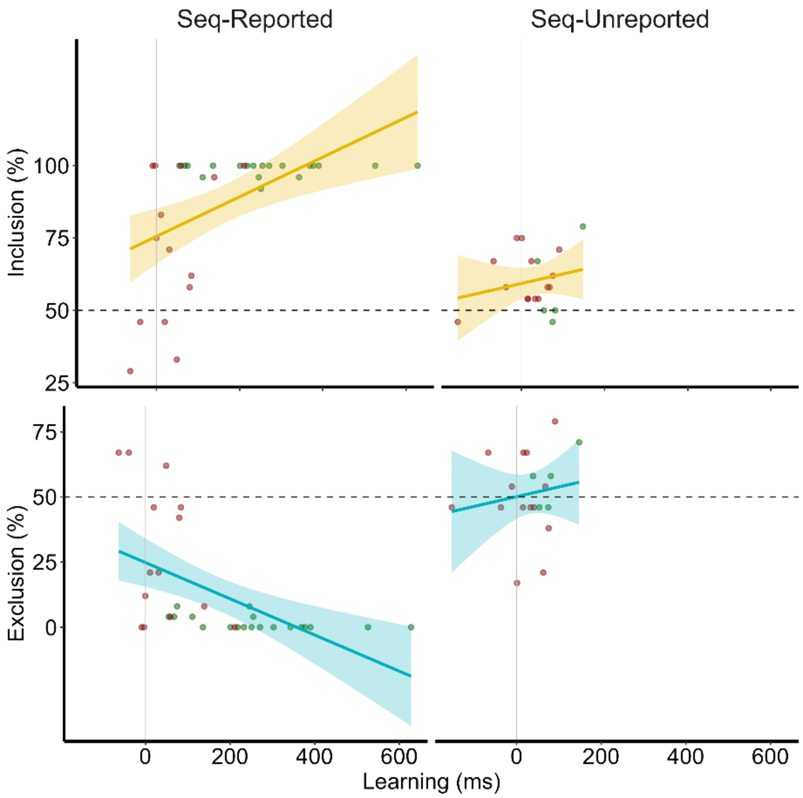

Supplementary Figure S4*. Correlation between PDP performance (percentage of grammatical target choice) and learning (estimated by the mean RT difference between Block 4 and Block 1), split by sequence report. Each dot represents a participant / condition (single, dual). Learner (dark green) and non-learner (dark red) refer to the results of sequence learning analyses performed at the individual level (see main text).*
